# Impact of climate change on dentistry and oral health: a scoping review

**DOI:** 10.1038/s41405-025-00310-2

**Published:** 2025-03-31

**Authors:** Upendra Singh Bhadauria, Bharathi Purohit, Nicolas Giraudeau, Mansi Atri, Harsh Priya

**Affiliations:** 1 Division of Public Health Dentistry, CDER-AIIMS, New Delhi, India; 2 CEPEL, CNRS, University of Montpellier, Montpellier, France; 3 ESIC Dental College & Hospital, Rohini, Delhi, India; 4 Division of Public Health Dentistry, Centre for Dental Education and Research, AIIMS, New Delhi, India

**Keywords:** Health care, Dentistry

## Abstract

**Background:**

Direct effects of climate change on different domains of general health have been well documented with evidence-based literature; however, the implications for oral health and dentistry have been addressed in different forms of research papers and lack a comprehensive evaluation.

**Objectives:**

We aimed to conduct a scoping review of the existing literature to elucidate the connections between the impact of climate change on oral health and dentistry, exploring how environmental shifts can influence dental diseases and practices and offering insights for future dental care using a systematic search strategy.

**Methods:**

A systematic search was carried out using keywords in PubMed, Embase, and Scopus databases. Boolean operators were also used to combine the searches and elaborate the search strategy. We did not apply any restriction of time frame or language to the articles.

**Summary of findings:**

A total of 10 papers were included in the final review. The findings from different papers have reported direct/indirect associations of climate change with oral diseases and conditions such as dental caries, dental erosion, and oral cancer; developmental defects of enamel; early childhood caries; periodontal disease; and dental trauma, skeletal, and dental fluorosis.

**Discussion:**

The findings synthesize a nascent yet significant body of research exploring how environmental changes driven by climate change impact the dental profession and oral health outcomes. Continued research and policy attention are imperative to address the complex and evolving challenges posed by climate change to oral health.

## Introduction

Climate change, characterized by rising global temperatures, altered weather patterns, and increased frequency of extreme weather events, is a pressing global issue with wide-ranging impacts on health. The Sixth Assessment Intergovernmental Panel on Climate Change (IPCC) report has confirmed a 1.09 °C rise in the global surface temperature with the global mean sea level rising by 0.2 m over the period 1901–2018 [1]. Even the findings from the Global Risk Report 2024 suggest that the impact of climate change on everyone is expected to worsen over the next decade [2].

While the direct effects of climate change on different domains of general health have been well documented with evidence-based literature [3, 4], the implications it has on oral health and dentistry, though addressed in different forms of research papers, lack a comprehensive, exhaustive evaluation.

Climate change can also exacerbate socioeconomic inequalities, impacting access to dental care. Disadvantaged communities, already at higher risk for poor oral health, may face greater challenges in accessing dental services due to the financial and logistical impacts of climate-related disruptions. Climate change also necessitates a shift in public health priorities, which can influence funding and focus for dental health initiatives. There is a risk that oral health could become inconsequential in the face of more immediate climate-related health threats, reducing the attention and resources dedicated to preventive and corrective dental care.

Additionally, it has also been said that the impact of climate change on oral health is multidimensional; it can be direct, indirect, or remotely associated and extensive [5]. While different opinions have been reported regarding the impact of climate change on dentistry and oral health, this study was aimed at conducting a scoping review of the existing literature on the impact of climate change on dentistry and oral health.

## Methods

A systematic search was carried out using key words such as “Climate change”, “Climate changes”, “Climate control”, “Climate controls”, “change, climate”, “changes, climate” “Oral Health”, “Dental Health”, “Dental Care”, “Oral Diseases”, “Dentistry”, “Dental Professionals” in PubMed, Embase, and Scopus databases. Boolean operators were also used to combine the searches and elaborate the search strategy. We did not apply any restriction of time frame or language to the articles.

Eligibility Criteria: All the papers that assessed the relationship/impact of climate change and oral health without any restriction of time frame/ language/ study design were included in this study. Papers that reported on the impact of oral health/dentistry on climate change or those assessing the impact of climate change on anything apart from oral health and dentistry were excluded from the study.

Screening Process: A systematic approach for study selection was carried out to screen the articles for eligibility and evaluate the titles and abstracts. We manually removed the duplicates, and the full-text articles were subsequently selected for analyzing data. The discrepancies arising during this stage were resolved by the third author. The inter-rater reliability across the title/abstract and full text review stages was assessed using Cohen’s Kappa and found to be 0.89 and 0.86, respectively. The data extracted from the included articles is presented in Table 1. Details regarding the primary author, year of publication, title, type of paper, and summary/recommendation were reported.

**Table 1:** Characteristic table for all the included studies.

Author	Year	Title	Type of paper	Country of primary author	Summary/Recommendations
Khanna	2010	Climate Change & Oral Health: Current Challenges & Future Scope	Review	India	Highlights the burden of oral precancer and cancer among the younger generation due to the rampant usage of tobacco, associated products and exposure to various amounts of solar radiation. This review recommends that the epidemiological assessment & readiness to tackle health burden arising due to change in climatic condition needs to be strengthened. Heath education and preventive programmes need to be targeted towards the vulnerable population. The public health infrastructure needs to be strengthened to adapt to the rapidly changing disease profile. Therefore, concerted efforts have to be made in order to understand these modifying factors.
Grose et al.	2016	Exploring attitudes and knowledge of climate change and sustainability in a dental practice: A feasibility study into resource management	QualitativeInterview Study	England	A qualitative approach was used to explore the knowledge and attitudes of staff in dental practice towards sustainability, and their understanding of reduce, reuse, and recycle approaches to resource use. This study highlights that climate change will negatively affect health service provision, warns that resources may become scarce due to extreme weather events causing supply and transport problems, which may cause costs in dentistry to rise. Suggests that dentists need to plan for resilience against reduced resources, highlights the financial benefits to efficient and environmentally safe use of resources and rationalization of waste.
Kemoli	2019	Paediatric oral health and climate change	Editorial	Kenya	Emphasizes the changes in the oral health associated with these climate changes, example oral cancers, increasing cases of dental development defects (DDE), changes in the pattern of early childhood caries, with periodontal disease. Addresses that the chemicals, in water, whether naturally occurring or introduced by human activities, can have a huge impact on teeth and oral mucosa of a child. Water with high quantities of fluoride can lead to skeletal and dental fluorosis. Arsenic from fertilizers can have adverse effects on the oral health of children.
Ajit	2020	Effects of climate change on oral health	Editorial	India	Highlights the role of poor air quality, food/water insecurity and social factors and their impact on oral health and diseases.
Ajit	2021	Effects of Global Climate Change on Oral Health: Overview	Commentary	India	Highlights the role of declining air quality, unsubstantial distribution of food and water and global warming and subsequent weather extremities.
Hackley	2021	Climate Change and Oral Health	Commentary	The United States of America	Provides an overview of the impact of climate change on oral health. Informs regarding the major risks of climate change by way of: Heat stress, poor air quality, food/water insecurity, extreme weather events, vector-borne illnesses and social factors. Provides an overview of the potential oral health associations to be researched in accordance with the associated health risks. Recommends developing strategic plans utilizing teledentistry, establishing strategies for personal and professional financial security, considering in office medication storage, avoiding antibiotic over usage.
Qamar and Qayum	2023	Understanding the Impact of Climate Change on Oral Health in Lower Middle-Income Countries	Letter to the editor	Pakistan	Reports that dental problems may become more prevalent because of poor dental hygiene and sanitation brought on by erratic supply of water imposed upon by climatic imbalance. Disasters and climate changes can result in dental trauma, including tooth fractures and avulsions.
Deshmukh et al.	2023	Climate Change on Oral Health and Dentistry: Association and Mitigation	Review	India	Aims to assess the impact of global climate change on Oral health outcomes and recommend measures to counter the looming crisis. Enumerates the ways through which climate change can affect oral health such as rising global temperatures, imbalance in food, diet and nutrition, contamination of air due to the greenhouse emissions, ozone depletion, water crises - floods and droughts, advent of newer and resurgence of old infectious communicable diseases and socio-economic effects. Recommends familiarization of oral health professionals towards the impact of climate change and its multidimensional effects on the oral health and sensitizing continuing education programs and training to health professionals towards adopting and advocating climate friendly practices.
Patil	2023	Addressing the impact of the climate crisis on oral health	Review	India	Addresses the increased risk of dental caries, oral cancer, periodontal disease, dental erosion and systemic diseases associated with climate change.
Folayan et al.	2024	Early childhood caries, climate change and the sustainable development goal 13: a scoping review	Scoping Review	Canada	Maps the published literature for existing evidence on the association between the Sustainable Development Goal (SDG) 13 and early childhood caries (ECC). Six papers were identified. four studies were linked to SDG 13.1 and they suggested an increased risk for caries with climate change. Two studies were linked to SDG 13.2 and they suggested that the practice of pediatric dentistry contributes negatively to environmental degradation. One study provided evidence on caries prevention management strategies in children that can reduce environmental degradation. The findings suggested that context specific and inter-disciplinary research is needed to generate evidence for mitigating the negative bidirectional relationships between SDG13 and ECC.

### Summary of findings

#### Literature search

**Fig. 1:**
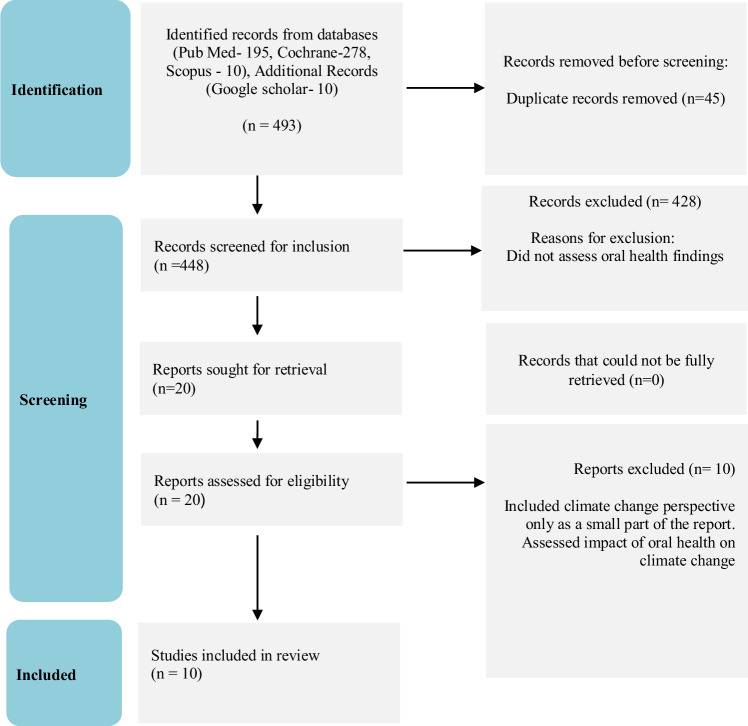
Flowchart of records screening.

The databases PubMed (195), Embase (278), Scopus (10), and additional sources (10) initially led to the identification of 493 records. 45 records were initially removed as they were found to be duplicates. 428 records that did not meet the inclusion criteria were excluded after screening the titles and abstracts. 20 papers remained, of which ten papers either reported the relationship of oral health/dentistry on climate change or included the impact of climate change only as a very small part of the report and were excluded, and thus finally 10 papers were included in the final analysis (Fig. 1) [5–14].

#### Study characteristics

The characteristics of the included study have been presented in Table 1. The papers included in this review were carried out between 2010 and 2024. Of the included papers, the primary authors of five papers were from India, one from England, one from the United States of America, and the remaining from Pakistan, Canada, and Kenya, each. With regards to the type of papers, four papers were review papers; including a scoping review; three were letters to editor (Editorial), and two were commentary/comments, while the remaining one was qualitative.

The role of poor air quality, food/water insecurity, extreme weather events, vector-borne illnesses, and social factors on oral health has been reported in a number of papers. Findings from multiple papers have revealed the importance of health education and preventive programs in addressing the challenges imposed by climate change.

The findings from different papers have reported direct/indirect associations of climate change with oral diseases and conditions such as dental caries, dental erosion, and oral cancer; developmental defects of enamel; early childhood caries; periodontal disease; dental trauma; skeletal and dental fluorosis. The association of these diseases highlights the gravity of the potential impact of climate change on oral health and dental diseases. Additionally, studies have also highlighted the negative impact of climate change on health service provision, warns that resources may become scarce due to extreme weather events causing supply and transport problems, which may cause costs in dentistry to rise.

## Discussion

This study aimed to synthesize existing literature on the intersection between climate change and dentistry and oral health through a scoping review. The findings synthesize a nascent yet significant body of research exploring how environmental changes driven by climate change impact the dental profession and oral health outcomes. This study is an amalgamation of different forms of papers providing a one-place assessment of the status and prospects for future research on the impact of climate change on the dental profession and oral health outcomes. Key themes identified include the direct effects of extreme weather events, the indirect effects of climate-induced socio-economic changes, and the broader implications for dental public health systems.

The findings from this review reported that extreme weather events such as hurricanes, floods, and heat waves can disrupt access to dental care and increase the prevalence of oral injuries. The impact of solar radiation and the burden of oral cancer have also been highlighted in a review [2]. Contaminated water supplies have also been attributed to the risk of dental infections and diseases such as dental caries and periodontitis due to poor oral hygiene and limited access to dental care facilities during and after such events [12]. Extreme weather conditions have also been associated with supply and transport problems, which may cause costs in dentistry to rise [7].

Indirectly, how climate change affects oral health has also been reported which may be through socio-economic disruptions. The imbalance in food and diet, in turn, impacts nutritional intake which is critical for maintaining good oral health [5, 15]. The literature also points to increased stress and mental health issues related to climate change as factors that can contribute to poorer oral hygiene practices and higher incidence of conditions like bruxism, which may further lead to temporomandibular joint disorders. Furthermore, the climate change may also exacerbate health inequalities, disproportionately affecting vulnerable populations such as communities from low-income groups, remote areas, and indigenous groups. The indigenous group often has limited access to dental care [16] and can be more susceptible to the adverse effects of climate change, exacerbating existing disparities in oral health outcomes.

The findings from this review report that sustainable procurement in professional ability may also be of importance to make a positive environmental impact. Additionally, emphasis on the utilization of a circular economy to regulate plastic use in which the products are maintained and used at the highest-value application for as long as possible can also serve as a sustainable solution [1].

Dental public health systems should be made more resilient to withstand and recover from the impacts of climate-related events. This includes developing emergency response plans that ensure the continuity of dental care services during disasters and foster community-based initiatives that promote oral health resilience.

The need for developing strategic plans utilizing teledentistry, establishing strategies for personal and professional financial security, and considering in-office medication storage may also be recommended to avoid antibiotic overusage [11]. Funding for research into the oral health impacts of climate change should be prioritized to better understand the risks and develop effective mitigation and adaptation strategies.

### Gaps in the literature and future research directions

Despite the need for growing evidence and a potential area to explore, there are significant gaps that remain in literature. There is a lack of epidemiological studies that can provide deeper insights into the long-term impacts of climate change on oral health. Additionally, more research is needed on the effectiveness of various adaptation strategies in mitigating the oral health impacts of climate change. Future research can include the following:

- The epidemiological studies investigating the direct and indirect effects of climate change on the prevalence and severity of oral diseases.
- Health Impact Assessments: Assessing the impact of climate-induced changes in water quality, food security, and socioeconomic conditions on oral health outcomes.
- Intervention Strategies: Developing and evaluating interventions to mitigate the impact of climate change on oral health, such as sustainable dental practices, emergency preparedness plans, and public health initiatives.
- Policy Analysis: Examining the effectiveness of policies aimed at addressing the intersection of climate change and oral health and identifying best practices for integrating oral health into climate change adaptation and mitigation efforts.
- Digital oral health: Potential areas of research can also include the impact of tele dentistry with regards to carbon emissions from other sources.

## Conclusions

The findings of the present study synthesize the impact of climate change and reviews the challenges it poses for maintaining oral health. Continued research and policy attention are imperative to address the complex and evolving challenges posed by climate change to oral health.

## Data Availability

The data that support the findings of this study are available from the corresponding author on reasonable request.
